# Supplementary material for: Neural oscillatory characteristics of feedback-associated activity in globus pallidus interna
Source: Sci Rep. 2023 Mar 13;13:4141. doi: 10.1038/s41598-023-30832-4 (PMC10011395; doi:10.1038/s41598-023-30832-4)
Supplement: Supplementary file 1 — Supplementary Information. [file 41598_2023_30832_MOESM1_ESM.pdf]

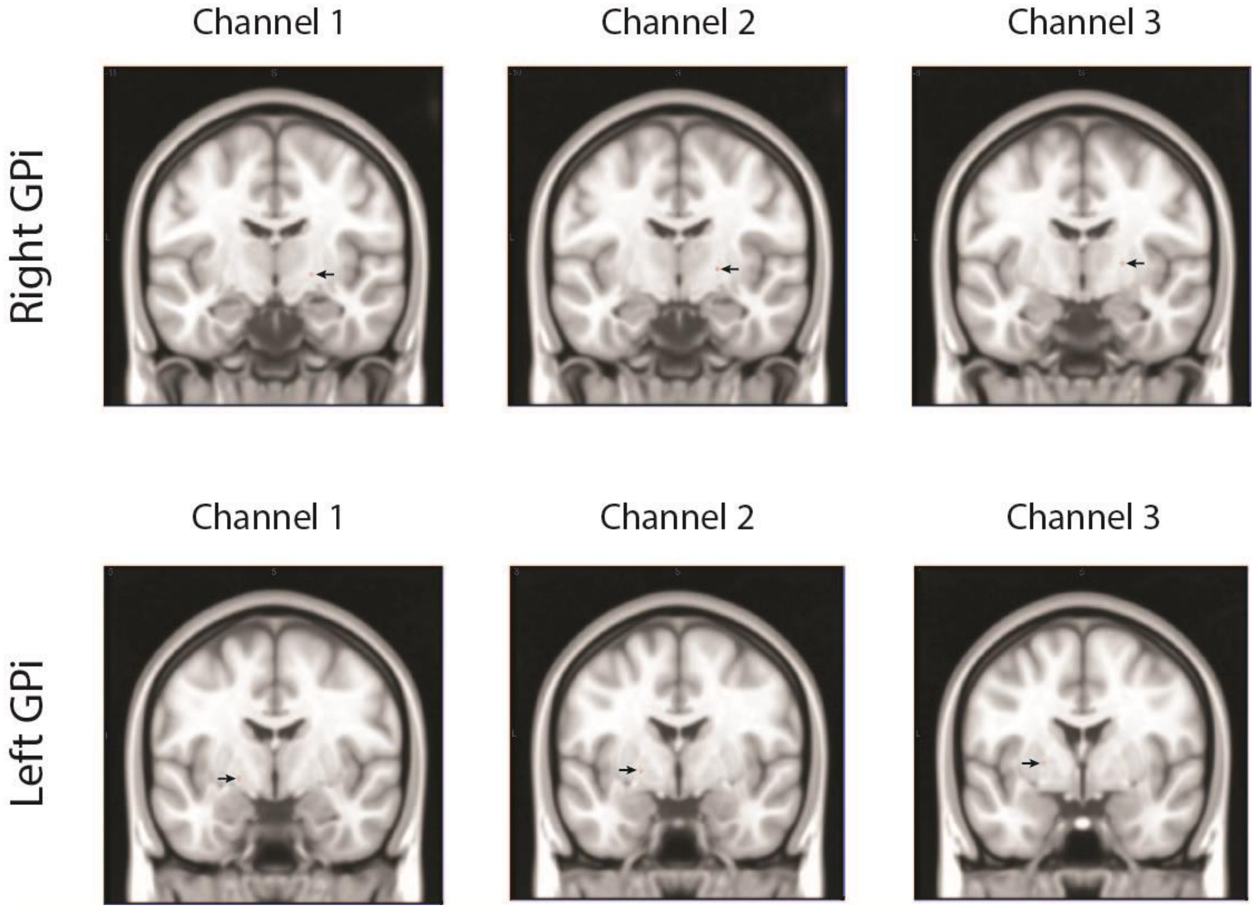

Supplementary Figure 1. MRI images of approximate DBS lead channel positions in PD and Dystonia patients. Lead positions were similar between patients. The images are in MNI space. Black arrows and red dots point to the channel positions.
